# The Role of Glutamate Metabolism and the GABA Shunt in Bypassing the Tricarboxylic Acid Cycle in the Light

**DOI:** 10.3390/ijms252312711

**Published:** 2024-11-26

**Authors:** Alexander T. Eprintsev, Galina B. Anokhina, Zakhar N. Shakhov, Polina P. Moskvina, Abir U. Igamberdiev

**Affiliations:** 1 Department of Biochemistry and Cell Physiology, Voronezh State University, 394018 Voronezh, Russia; bc366@bio.vsu.ru (A.T.E.); dowi2009@mail.ru (G.B.A.); zakharshakhov@gmail.com (Z.N.S.); polinamoskvina2001@gmail.com (P.P.M.); 2 Department of Biology, Memorial University of Newfoundland, St. John’s, NL A1C 5S7, Canada

**Keywords:** GABA shunt, glutamate, glutamate dehydrogenase, phytochrome, tricarboxylic acid cycle

## Abstract

Glutamate is an essential amino acid in both the energy and biosynthetic processes in plant cells. The aim of this work was to study changes in glutamate metabolism upon irradiation of maize (*Zea mays* L.) leaves with light of different spectral compositions, as well as to identify mechanisms regulating the work of enzymes involved in the studied process. A study was conducted of light-induced changes in glutamate metabolism in maize leaves, mediated by redirecting the glutamate flow to the γ-aminobutyric acid (GABA) shunt. Glutamate dehydrogenase (GDH) was more active in darkness, and the irradiation by red light inhibited the expression of both the *Gdh1* and *Gdh2* genes. EGTA and ruthenium red abolished the effects of light, indicating the participation of Ca^2+^ ions in phytochrome signal transduction. Contrary to GDH, glutamate decarboxylase (GAD) activity was moderately higher in the light, stimulated by red light, while far-red light reversed the effect. The effect of light on *Gad* expression was more pronounced than on GAD activity. Irradiation by red light also resulted in the increase in activity of GABA transaminase (GTA), which was abolished by far-red light. The third enzyme of the GABA shunt, succinic semialdehyde dehydrogenase (SSADH), was also activated by light. The effect of light on the expression of *Ssadh1*, but not on *Ssadh2*, was phytochrome-dependent. It is concluded that irradiation by light shifts glutamate metabolism from GDH to GAD with the activation of GABA transaminase and SSADH. This suggests that the GABA pathway plays a role in the maintenance of the tricarboxylic acid cycle in the light via bypassing its reactions when the 2-oxoglutarate dehydrogenase complex is inhibited and the cycle switches to the open mode.

## 1. Introduction

The transitions from darkness to light and changes in light intensity are fundamental for the survival of plants. The ability to perceive light of different wavelengths and intensities activates several signal transduction pathways, which in turn regulate plant growth, physiology, morphology, and immunity [1]. In addition, photosynthetic reactions themselves regulate biochemical mechanisms in plant tissues [2]. This is evidenced by the fact that in Arabidopsis and other studied plants, a number of genes are transcriptionally induced by circadian rhythms [3]. Higher plants possess several families of photoreceptors that can detect light ranging from UVB to far-red [4,5]. Due to the differences in the light spectrum, intensity, direction, and photoperiod, a number of photoreceptors, including phytochrome, cryptochrome, and phototropin, have evolved to adapt plants to different light conditions [6]. Functional specialization within photoreceptor families led to the emergence of photoreceptors that are capable of detecting light over a wide range of wavelengths and intensities. Genetic and photobiological studies conducted on *A. thaliana* plants have shown that these light sensors mediate numerous adaptive responses (e.g., phototropism and shade avoidance) and developmental transitions (e.g., germination and flowering). Some physiological responses are specialized to be triggered by a specific photoreceptor; however, often multiple photoreceptors are known to mediate a coordinated response. *A. thaliana* exhibits cross-responses to various environmental stressors, which may be mutually exclusive and/or substitutive, e.g., between light and temperature, or light and pathogens [1].

Phytochrome is an important class of photoreceptors that sense red and far-red light [7,8,9]. To date, five types of phytochromes have been identified in Arabidopsis (phyA, phyB, phyC, phyD, phyE), which have two photo-interconvertible forms [10,11]. Upon exposure to red light, the conformation of phytochromes allosterically changes from an inactive form that absorbs red light to an active form that absorbs far-red light. The inactive form is located in the cytosol, whereas the active form is translocated to the nucleus [10,11], where it interacts with a variety of factors to regulate the transcription of target genes and mediate subsequent photoreactions [11]. Phytochrome interacting factors (PIFs) primarily act as negative regulators of photomorphogenesis in response to light. They also regulate many pathways and processes, including anthocyanin biosynthesis, thermomorphogenesis, humoral signaling, and responses to biotic and abiotic stresses by interacting with a variety of cellular molecules [12]. In maize (*Zea mays*), there is evidence that the *ZmPIF1* and *ZmPIF3* genes can increase drought tolerance by causing stomatal closure [13,14]. In the PIF4 mutant of Arabidopsis, hypocotyl growth is insensitive to high temperatures, indicating a key role of *AtPIF4* in thermoresponsive plant growth. Thus, PIF is considered a hub for the integration of environmental and hormonal signaling pathways [12].

The scheme of light signal transduction with the participation of phytochromes can be represented as follows [15,16,17]:

Reception of light quanta by phytochrome chromoproteins → conformational transformations of phytochrome → opening/closing of ion channels including Ca^2+^
channels → translocation of chromoproteins into the nucleus → interaction of chromoproteins with transcription factors with their subsequent interaction with genes → activation/inhibition of gene transcription → biological effect

The physiological action of phytochrome spans the cytoplasm and the nucleus. In the cytoplasm, red light controls Ca^2+^ transport and its absorption by the cell [18]. Among the transport systems that maintain a constant level of Ca^2+^ in the cell, Ca^2+^-ATPase is under the control of phytochrome. Light causes conformational changes in phytochrome molecules, which, moving into the nucleus, cause the activation of ATP-dependent calcium channels, which results in Ca^2+^ movement from the cytoplasm to the nucleus. In addition, an increase in the Ca^2+^ concentration regulates the cGMP level [19]. Phytochrome controls the cGMP content in the cytoplasm via guanylate cyclase [20]. Ca^2+^ acts as a secondary messenger—an information carrier—thus being located between the receptor and reactive parts of various cell signaling systems. Calmodulins (CaMs) are proteins that are among the most common sensory proteins and are found in the apoplast, cytosol, endoplasmic reticulum, and nucleus. CaMs, with the participation of calcium, interact with various target proteins [21], which participate in various physiological processes in plant cells, including transport, cytoskeletal rearrangements, cell division, and gene expression [22,23,24].

The light-induced transition of phytochrome to the active form is caused by a change in the calcium concentration gradient due to calcium transport from the cytoplasm to the nucleus, which in turn causes a calcium–calmodulin-dependent cascade of reactions that promotes changes in the transcription of various genes, including genes for TCA cycle enzymes [17,25]. It is currently known that the activity of a number of enzyme systems is regulated with the participation of the calcium–calmodulin-dependent system. Regulation of glutamate decarboxylase (GAD) activity by calcium is carried out through the calmodulin (CaM)-binding domain. Ca^2+^/CaM binding eliminates dissociation of the oligomer encoded by the *AtGAD1* gene. The N-terminal domain of this protein plays a key role in maintaining the oligomeric form, since the removal of the first twenty-four N-terminal residues significantly affects oligomerization due to the formation of the dimeric form of the enzyme [26]. Positive regulation by Ca^2+^ and CaM, as shown in the studies of Snedden et al. [27], occurs through a 2.4-fold stimulation of *V*_max_ and a 55% decrease in *K*m. Regulation by the Ca^2+^/CaM-coupled system may reflect the need for rapid modulation of GAD in response to external signals [28]. Thus, the Ca^2+^/CaM-dependent system is involved in regulating the operation of the entire GABA shunt by regulating the catalytic activity of GAD [29]. The activity of several other enzymes of respiratory metabolism is regulated by phytochrome via a calcium-dependent system [25,30]. In plants, the tricarboxylic acid (TCA) cycle operation in the light is partially inhibited when the photosynthetic system takes on the main role in providing the cell with energy [31,32,33,34]. Most of the mitochondrial enzymes that participate in the TCA cycle, such as succinate dehydrogenase (EC 1.3.99.1), malate dehydrogenase (EC 1.1.1.37), aconitate hydratase (EC 4.2.1.3), citrate synthase (EC 2.3.3.1), fumarase (EC 4.2.1.2), and 2-oxoglutarate dehydrogenase (2-OGDH; EC 1.2.4.2), are more active in darkness and suppressed in the light [35,36,37,38,39,40]. The use of specific inhibitors, succinylphosphonate and carboxyethyl ether, confirmed the inhibitory effect of light on the operation of the 2-OGDH complex [41]. A decrease in the respiratory rate is associated with changes in the regulation of metabolic and signaling pathways, which leads to an imbalance of carbon–nitrogen metabolism and cellular homeostasis. The inducible changes in primary metabolism have been associated with modulations in the expression of genes belonging to amino acid biosynthesis, plant respiration, and sugar metabolism [42,43,44]. Oxidation of 2-oxoglutarate (2-OG) by the 2-OGDH complex strongly affects the distribution of TCA cycle intermediates and the operation of the γ-aminobutyric acid (GABA) shunt. The TCA cycle apparently functions in a non-cyclic manner upon the inhibition of the 2-OGDH complex during the photoperiod [31,34,41,45,46].

In illuminated leaves, the intensity of decarboxylation in the TCA cycle can decrease by up to 80%, and the decarboxylation reaction carried out by pyruvate dehydrogenase is inhibited by 30% compared to dark respiration [31]. In photosynthetic tissues, the activity of the complete citric acid cycle appears to be reduced, and the non-cyclic pathway is likely more important in illuminated leaves due to the transport of organic acids from mitochondria [34,45]. There is strong evidence that the operation of the TCA cycle in illuminated leaves provides 2-OG for ammonium assimilation and for the reactions of secondary metabolism [46,47]. The intensity of nitrogen assimilation influences respiration during the photoperiod [46]. An important role of glutamate dehydrogenase (GDH; EC 1.4.1.3) in adaptation [48] may indicate its participation in the regulation of metabolism by light. In the dark, GDH deaminates glutamate to 2-OG, which is supplied to the TCA cycle for additional energization of the cell. This pathway is essential under conditions of carbohydrate starvation during prolonged incubation of plants in the dark. The discovery of the receptors of GABA and glutamate in plants has led to greater evidence that GABA also plays a role as a signaling molecule in plants [49,50]. However, very little is known about the participation of GABA in the transmission of light signals.

Not only does the TCA cycle itself undergo significant functional changes depending on the light regime but high-intensity daylight and UV radiation are known to induce the GABA shunt activity and GABA accumulation as well [51,52]. In plants, two control points of GABA shunt regulation have been described: positive regulation of GAD by the Ca^2+^/CaM system in the cytoplasm and negative regulation of SSADH by ATP and NADH in mitochondria. The Ca^2+^/CaM-dependent system is involved in the regulation of the entire GABA shunt by affecting the catalytic activity of GAD [29]. Experiments with the mutants for the genes encoding SSADH have demonstrated the important role of this enzyme in protecting plant cells from UV rays: UV-B had the most adverse effect, while light from the photosynthetic active range had a much lower effect. UV light in mutant plants caused a rapid increase in the levels of hydrogen peroxide and reactive oxygen species [53]. It was demonstrated that blue light activates GABA transaminase in tomato [54]. There is evidence that the GABA shunt activation in illuminated leaves of higher plants is caused by a decrease in the activity of the 2-OGDH complex, and in these conditions, the GABA bypass pathway regenerates succinate via succinic semialdehyde dehydrogenase (SSADH; EC 1.2.1.24) [55]. However, there are practically no data on the direct participation of the GABA shunt enzymes in the transmission of light signals.

In the current work, we studied the regulation by light of glutamate metabolism via the phytochrome control of GDH and of the enzymes of the GABA shunt. We showed that GDH is inhibited by light via the phytochrome mechanism, while the GABA shunt is stimulated in the light, apparently compensating for the inhibition of several reactions of the TCA cycle by the increased redox level. We demonstrated the participation of phytochrome in the transduction of the light signal to the genes of GAD, GABA transaminase, and SSADH. We conclude that the GABA shunt is an important compensatory bypass mechanism not only during the adaptation to different stresses but also in the adjustment of plant respiration to light conditions.

## 2. Results

### 2.1. Glutamate Dehydrogenase Activity

**Figure 1:**
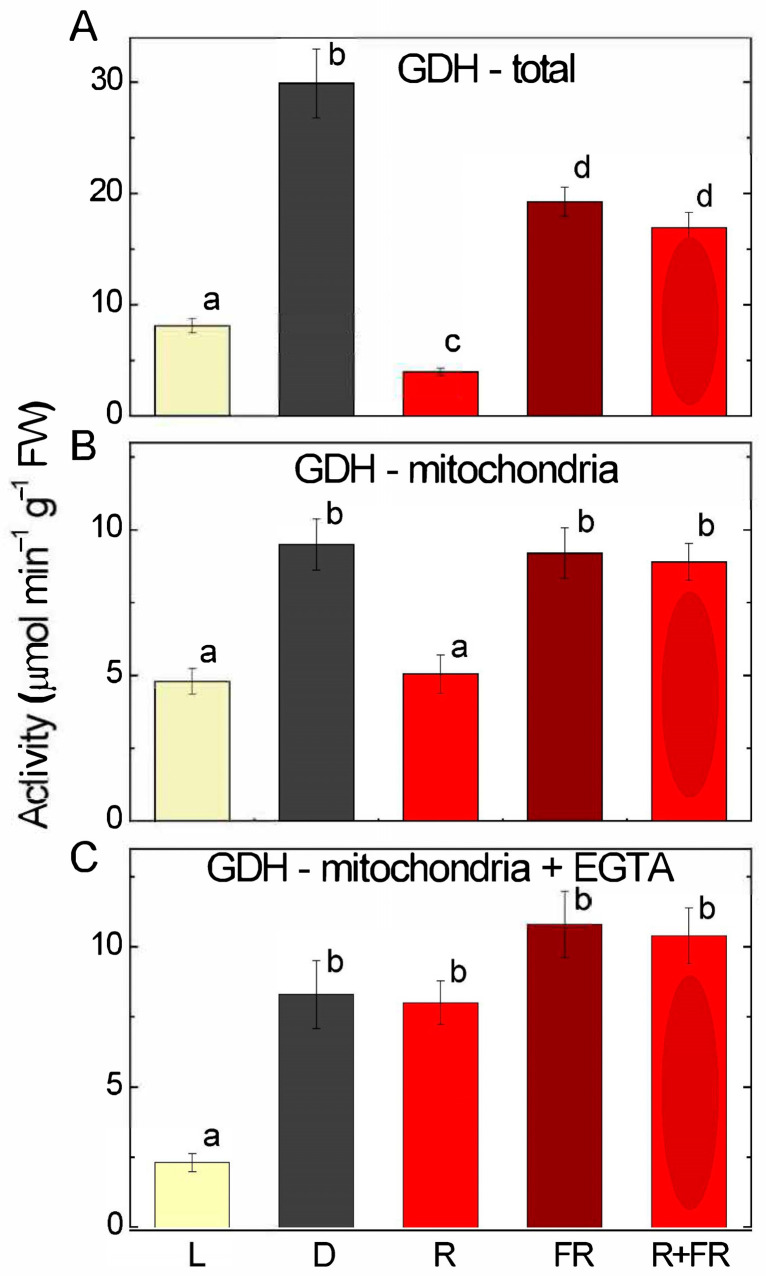
Glutamate dehydrogenase activity in maize leaves under different light conditions. (**A**)—the activity measured in crude extract; (**B**)—the activity measured in the mitochondrial fraction; (**C**)—the activity in the mitochondrial fraction of the plants treated with EGTA. L—light; D—dark; R—red light (660 nm); FR—far-red light (730 nm); R + FR—red light followed by far-red light. The results are presented as the average ± standard error of the mean (SEM). Differences were analyzed for statistical significance using Student’s *t*-test with Bonferroni correction for multiple comparisons. The letters indicate statistically significant differences at *p* < 0.05 (*n* = 5).

The study of the influence of light and its spectral composition on the operation of GDH in maize leaves revealed that in the plants exposed to light, the total activity of the studied enzyme was more than three times lower than in the plants kept in darkness (Figure 1A). Irradiation with red light (660 nm) decreased GDH activity by several times relative to the values observed in darkness. Irradiation with far-red light (730 nm) resulted in values of GDH activity of ~60% as compared to darkness; when seedlings were irradiated sequentially with red light and far-red light, the activity remained at the level observed upon irradiation with far-red light.

Analysis of the GDH activity in the isolated maize leaf mitochondria showed a similar, although less striking, pattern of GDH inhibition in the light and upon irradiation with red light (Figure 1B). In darkness, the values of enzymatic activity were almost twice as high as in light. Maize seedlings exposed to far-red light for 15 min showed similar levels of GDH activity in mitochondria as in darkness. The inhibitory effect of red light was abolished in the sequential irradiation with red and far-red light.

The use of EGTA, a chelating agent with a high affinity for Ca^2+^, made it possible to identify the effect of free calcium cations on the transmission of the light signal. The treatment with EGTA resulted in the removal of the inhibitory effect of red light (Figure 1C). A similar effect as with EGTA, although less pronounced, was observed in the experiments, where ruthenium red, acting as a blocker of calcium channels, was applied (Supplementary Figure S1A).

### 2.2. Glutamate Dehydrogenase Expression

**Figure 2:**
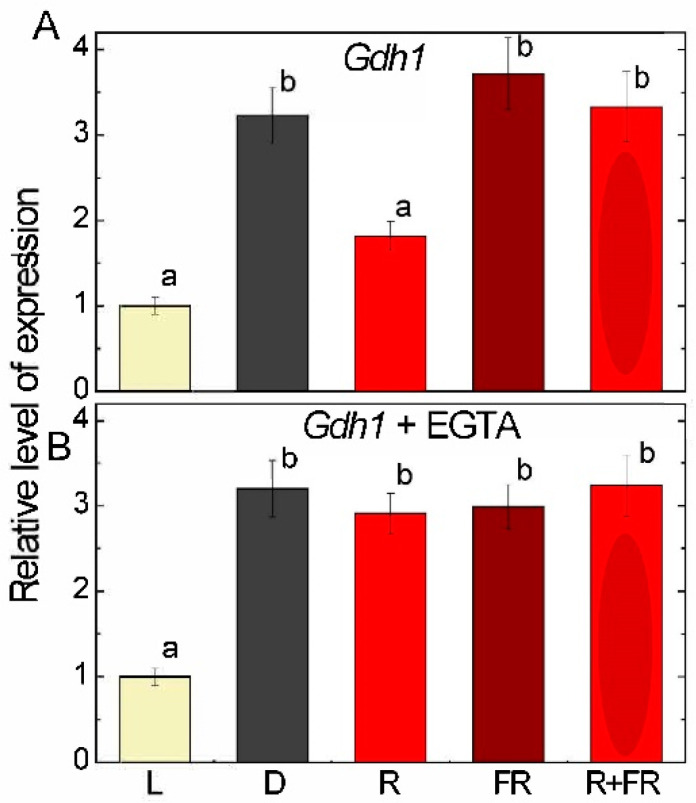
Expression of the glutamate dehydrogenase gene *Gdh1* in maize leaves under different light conditions. (**A**)—expression without EGTA; (**B**)—expression after the treatment with EGTA. Abbreviations are the same as in Figure 1. The results are presented as the average ± standard error of the mean (SEM). Differences were analyzed for statistical significance using Student’s *t*-test with Bonferroni correction for multiple comparisons. The letters indicate statistically significant differences at *p* < 0.05 (*n* = 5).

Analysis of the relative level of transcripts of the *Gdh1* gene showed a marked decrease in mRNA transcripts in the light and upon irradiation by red light as compared to darkness. Far-red light irradiation of darkened plants did not cause significant changes in the transcriptional activity of the *Gdh1* (LOC542220) gene. Irradiation with far-red light after red light led to the elimination of the inhibitory effect of red light on the transcription of the *Gdh1* gene (Figure 2A). The treatment with EGTA abolished the inhibitory effect of red light (Figure 2B); the same pattern was observed for the plants treated with ruthenium red (Supplementary Figure S1B).

**Figure 3:**
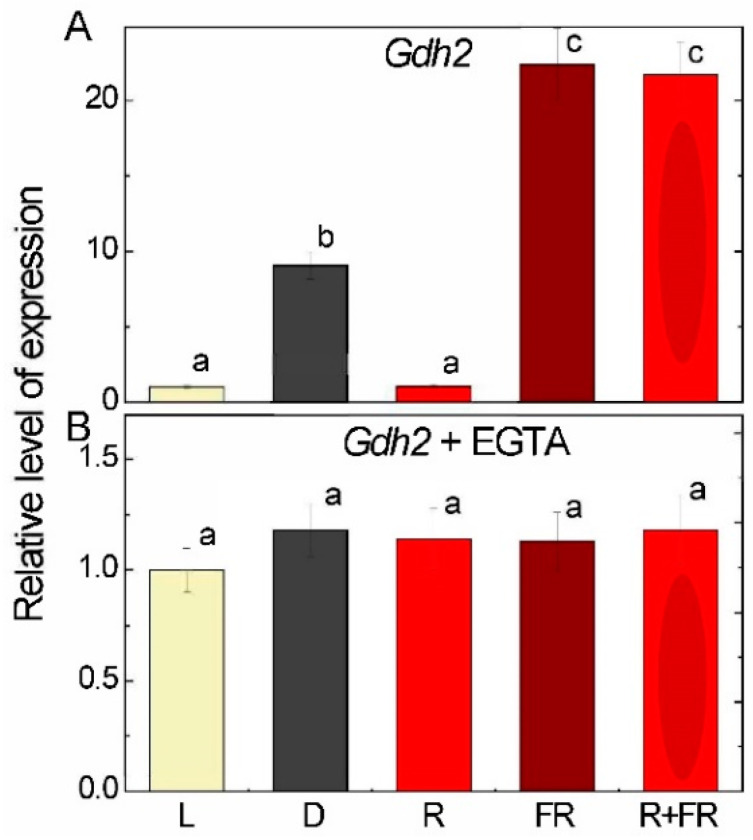
Expression of the glutamate dehydrogenase gene *Gdh2* in maize leaves under different light conditions. (**A**)—expression without EGTA; (**B**)—expression after the treatment with EGTA. Abbreviations are the same as in Figure 1. The results are presented as the average ± standard error of the mean (SEM). Differences were analyzed for statistical significance using Student’s *t*-test with Bonferroni correction for multiple comparisons. The letters indicate statistically significant differences at *p* < 0.05 (*n* = 5).

A study on the effect of light of different wavelengths on the expression of the *Gdh2* (LOC100193614) gene showed a severe inhibition of expression in the light and by irradiation of red light (Figure 3A). The levels of *Gdh2* gene expression were even more than twice as high upon irradiation by far-red light than in darkness, and they exhibited the same high level when far-red light was applied after red light. The treatment with EGTA abolished the inhibitory effect of light and of the irradiation by red light (Figure 3B). A similar effect was observed when the plants were treated with ruthenium red (Supplementary Figure S1C).

### 2.3. Glutamate Decarboxylase Activity and Expression

**Figure 4:**
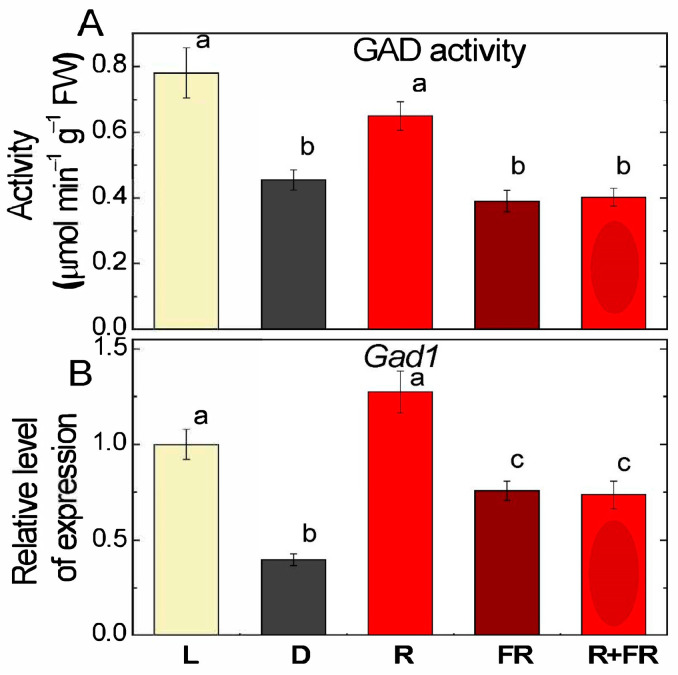
Glutamate decarboxylase activity (**A**) and expression of *Gad1* gene (**B**) in maize leaves under different light conditions. Abbreviations are the same as in Figure 1. The results are presented as the average ± standard error of the mean (SEM). Differences were analyzed for statistical significance using Student’s *t*-test with Bonferroni correction for multiple comparisons. The letters indicate statistically significant differences at *p* < 0.05 (*n* = 5).

The values of glutamate decarboxylase (GAD) activity in the plants exposed to light were almost twice as high than in darkness. Irradiation with red light caused an increase in GAD activity relative to the group of plants exposed in the dark, while irradiation with far-red light after darkness and after red light resulted in similar values as in darkness (Figure 4A).

Analysis of the relative level of transcripts of the *Gad1* (LOC100381655) gene showed that in darkness, its transcriptional activity was ~2.5 times lower than in the light, while irradiation by red light increased the concentration of *Gad1* transcripts to the level observed in the light (Figure 4B). In the plants irradiated by far-red light after darkness and after red light, a slight increase in the relative level of transcripts was observed relative to the values recorded in the plants incubated in darkness.

### 2.4. GABA Transaminase Activity and Expression

**Figure 5:**
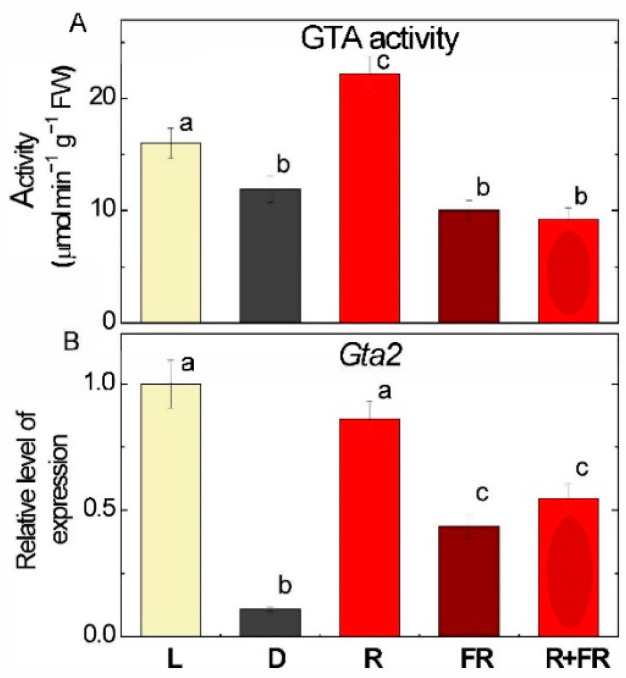
GABA transaminase activity (**A**) and expression of *Gta2* gene (**B**) in maize leaves under different light conditions. Abbreviations are the same as in Figure 1. The results are presented as the average ± standard error of the mean (SEM). Differences were analyzed for statistical significance using Student’s *t*-test with Bonferroni correction for multiple comparisons. The letters indicate statistically significant differences at *p* < 0.05 (*n* = 5).

Measurement of the catalytic activity of GABA transaminase (GTA) in maize leaves irradiated with light of different wavelengths demonstrated the light-dependent nature of the regulation of this enzyme (Figure 5A). In the leaves of plants exposed to light, GTA activity was slightly (1.2 times) higher than in the leaves of dark-growing plants. Irradiation with red light increased GTA activity by 1.6 times as compared with the values in darkness. Irradiation with far-red light after darkness and after red light resulted in activity levels comparable to those in darkness.

Analysis of changes in the transcription profile of the *Gta2* (*LOC103645944*) gene showed that light caused an almost 9-fold increase in the relative level of transcripts of the gene relative to the plants incubated in darkness (Figure 5B). Irradiation with red light also had an inducing effect on the transcriptional activity of this gene. The plants exposed to far-red light (applied after darkness or after red light) showed an intermediate concentration of the *Gta2* mRNA in leaves, which was lower than in the light, and higher than in darkness.

### 2.5. Succinic Semialdehyde Dehydrogenase Expression

**Figure 6:**
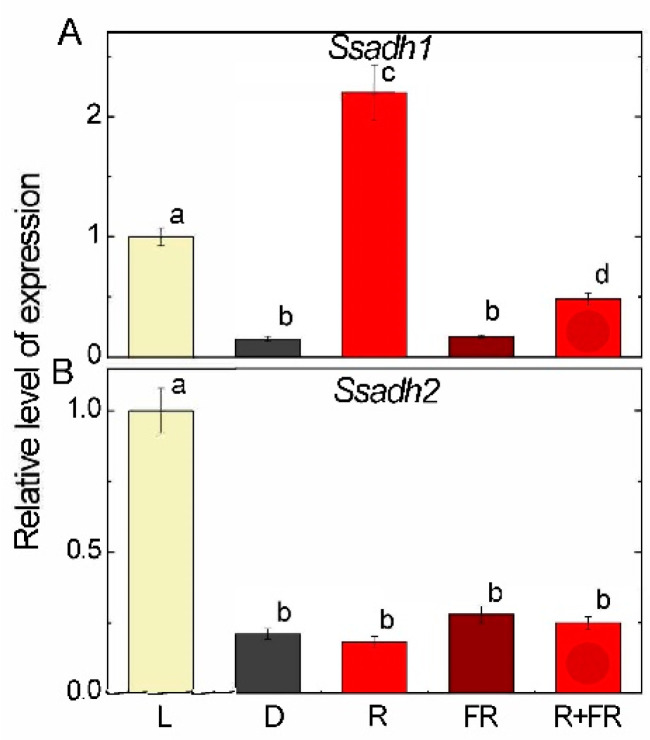
Expression of the succinic semialdehyde dehydrogenase genes *Ssadh1* (**A**) and *Ssadh2* (**B**) in maize leaves under different light conditions. Abbreviations are the same as in Figure 1. The results are presented as the average ± standard error of the mean (SEM). Differences were analyzed for statistical significance using Student’s *t*-test with Bonferroni correction for multiple comparisons. The letters indicate statistically significant differences at *p* < 0.05 (*n* = 5).

Measurement of the relative level of transcripts of the *Ssadh1* gene showed an almost 10-fold increase in the mRNA concentration of the studied gene when exposed to light as compared to darkness (Figure 6A). Upon irradiation with red light, the concentration of *Ssadh1* transcripts was more than 20 times higher than in darkness. Far-red light applied after darkness had no effect on changes in the mRNA level of the *Ssadh1* (*LOC100280779*) gene. When far-red light was applied after red light, a moderate increase in the transcriptional activity of the *Ssadh1* gene as compared to the dark and red light levels was observed.

Analysis of changes in the transcriptional activity of the *Ssadh2* (*LOC100284047*) gene upon irradiation of maize leaves with different wavelengths showed a different pattern as compared to the *Ssadh1* gene (Figure 6B). Exposure of seedlings to light caused an increase in the *Ssadh2* gene transcripts by five times as compared to the plants incubated in darkness. Irradiation with red, with far-red light, and with both did not significantly change the transcriptional activity of the *Ssadh2* gene.

## 3. Discussion

Irradiation of plants leads to conformational changes in the structure of the phytochrome molecule and to concentration changes in Ca^2+^ ions, as a result of which the active form of phytochrome moves to the nucleus, where it indirectly regulates (suppresses) the transcription of GDH genes through a system of transcription factors [11,12]. Previously, we showed that in the light and upon irradiation with red light, a significant decrease in 2-OGDH expression and activity is observed, which limits the rate of the entire TCA cycle [39]. GDH activity and expression increase in the dark and upon irradiation with far-red light (Figure 1, Figure 2 and Figure 3), which leads to the intensification of the operation of the TCA cycle via the supply of 2-OG derived from glutamate. These observations confirm the evidence that light suppresses the TCA cycle activity [31,39,40]. Our data are consistent with the results of experiments by Garnik et al. [56], who showed that the GDH activity in *A. thaliana* seedlings rapidly declines during the transition from dark to light within half an hour. During the transition from light to dark, the activity increases and reaches a maximum after 8 h incubation in the dark, and the expression of Arabidopsis *GDH1* and *GDH2* genes depends on the redox state of the plastoquinone pool [57]. Miyashita and Good [58] showed that Arabidopsis plants are more resistant to prolonged incubation in the dark than knockout mutants of the *GDH1* and *GDH2* genes. Based on these data, it was suggested that in plants, GDH represents a mechanism of adaptation of cellular metabolism to carbohydrate starvation due to the use of glutamate as an alternative energy source [58]. This is confirmed by the observation that soluble sugars affect the expression of GDH genes, e.g., treatment of 7-day-old Arabidopsis seedlings with 3% glucose solution led to a decrease in the relative level of *GDH1* and *GDH2* gene transcripts [59]. In the light, the photosynthetic system is the main source of energy for the plant cell. In this regard, the need for intensive functioning of the TCA cycle decreases and the activity of its enzymes is maintained at a lower level. Our results demonstrate that upon irradiation, the maintenance of the TCA cycle and of the mitochondrial electron transport occurs in part due to the operation of the GABA shunt. At the same time, the supplier of glutamate for the GABA shunt in the light is likely not GDH (which is inhibited), but the GS/GOGAT system of chloroplasts, which is active in photosynthetic tissues [34]. Activation of GAD, the first enzyme of the GABA shunt, in the light (Figure 4) can be related to the supply of glutamate from chloroplasts. Glutamate in the cytoplasm is decarboxylated into GABA, which is subsequently transported into mitochondria, where GABA is transaminated to form succinic semialdehyde due to light-induced transcription of the *Gta* genes encoding GABA transaminase (Figure 5). The activation of SSADH by induction of the *Ssadh1* gene in the light (Figure 6A) ensures the supply of succinate for the TCA cycle. The second gene *Ssadh2* is also induced in the light, but the mechanism of this induction does not involve phytochrome (Figure 6B). The obtained data suggest that the active form of phytochrome activates a Ca^2+^-dependent cascade of reactions, which leads to the induction of genes encoding the enzymes of the GABA shunt.

In illuminated leaves, the intensity of decarboxylation in the TCA cycle reactions significantly decreases [60], and the non-cyclic operation of the TCA pathway takes place due to the transport of organic acids from the mitochondria [31,34,45]. In these conditions, when the operation of the 2-OGDH complex is inhibited [39,46], the GABA shunt undergoes activation [61,62] due to the induction of the enzymes of GABA metabolism. GABA can act as an important source of succinate for the TCA cycle in illuminated leaves [55]. This is supported by experimental data obtained by Ludewig et al. [63], who showed that high-intensity daylight and UV radiation induce the functioning of the GABA shunt while promoting the accumulation of GABA. It was shown earlier that light affects nitrogen metabolism in different ways, e.g., in salt-stressed durum wheat, it inhibits the synthesis of glycine betaine [64], suppresses proline and glutamate accumulation, while the content of GABA, amides, and minor amino acids increases [51]. Araújo et al. [41] demonstrated that the inhibition of the 2-OGDH complex by phosphonate analogs that can mimic the suppression of the complex in the light leads to the switch to the GABA shunt.

The process of oxidation of 2-oxoglutarate to succinyl-CoA via the 2-OGDH complex occurs more intensively in the dark and is inhibited in the light, which is associated with the high level of photosynthetically formed ATP [65]. ATP inhibits 2-OGDH, which also leads to the suppression of the GDH in the direction of glutamate deamination via the feedback mechanism [66,67]. Moreover, high levels of NADH and ATP inhibit SSADH operation [29], and the increase in its expression in the light observed in this study can partly compensate for this suppression.

The mechanism of transduction of the light signal to the genes *Gdh1, Gdh2, Gad1, Gta2,* and *Ssadh1* described in this paper involves the control of the expression of these genes by the phytochrome system mediated by Ca^2+^ ions. From the results obtained in this study, it is seen that GDH activity and the expression of both *Gdh* genes are suppressed by light via the phytochrome mechanism, and the effect of phytochrome is mediated by Ca^2+^ ions (as seen from the incubation with EGTA and ruthenium red) (Figure 1, Figure 2 and Figure 3). Disruption of calcium transport caused by the use of EGTA and ruthenium red leads to the disruption of light signal transmission and thus to the absence of the inhibitory effect of red light on the transcription of *Gdh* genes and, as a consequence, on GDH activity. The *Gdh2* gene is inhibited by red light and white light much stronger than the *Gdh1* gene; its inhibition is more than 10-fold as compared to the 2–3-fold inhibition of *Gdh1*. Previously, it was shown [68] that the *Gdh2* gene encodes the subunits of GDH that more readily catalyze the reverse reaction of 2-OG amination. This means that while glutamate oxidation via GDH is partially suppressed in the light, glutamate formation from 2-OG by GDH is almost fully blocked; hence, glutamate should be formed in the light exclusively in chloroplasts via the GS/GOGAT system.

Glutamate in the light is readily decarboxylated with the formation of GABA, which is evidenced by the experiments on the measurements of GAD activity and on the expression of the *Gad1* gene. The effect is significant, especially for the expression of the *Gad1* gene, which is activated three times by white light and four times by red light (Figure 4). Even stronger is the activation of *Gta2* expression by red and white light (near 10-fold), although the changes in GTA activity are less pronounced (Figure 5). The observed differences in light effects can be explained by the fact that the regulation of transcription of the studied genes in response to light signal is a complex process and includes a Ca^2+^-dependent mechanism, transcription factors, etc.; in addition, changes in the transcription level of the *Gad1* and *Gta2* genes may be associated with the transport of GABA through the mitochondrial membrane [69,70,71,72,73,74,75].

The expression of both genes encoding SSADH is strongly activated by light, but the mechanisms are different, as seen from the study of the effects of red and white light (Figure 6). The gene *Ssadh1* is regulated via the phytochrome mechanism, with red light activation of this gene by more than 10 times as compared to darkness, while the gene *Ssadh2* is controlled by light independently from phytochrome effects. Red and far-red light were inefficient for this gene, which may indicate the participation of other mechanisms involving either cryptochrome or other light receptors.

**Figure 7:**
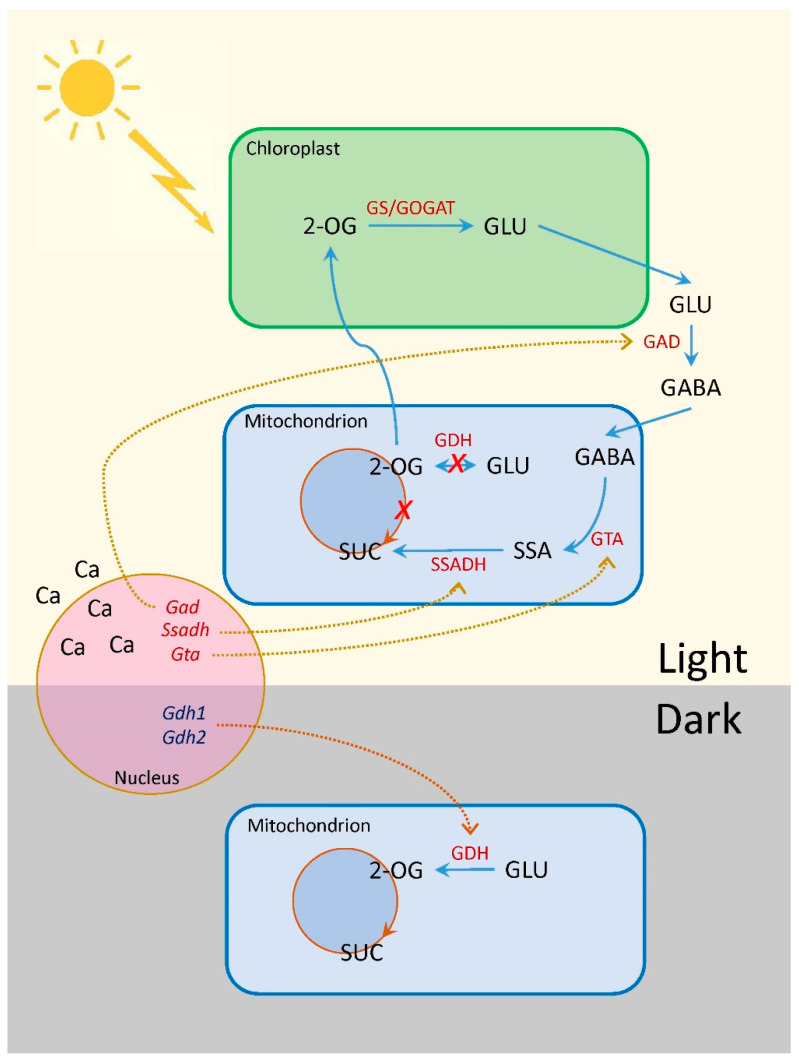
Changes in glutamate metabolism in darkness and light. Abbreviations: Ca, calcium ions; GABA, γ-aminobutyric acid; GLU, glutamate; GS/GOGAT, glutamine synthetase–glutamate synthase; 2-OG, 2-oxoglutarate; SSA, succinic semialdehyde; SUC, succinate. Enzymes: GAD, glutamate decarboxylase; GDH, glutamate dehydrogenase; GTA, GABA transaminase; SSADH, SSA dehydrogenase. Corresponding genes are given in italics. The red-colored *X* indicates the suppression of the reaction in the light.

The obtained results demonstrate that the GABA shunt provides the maintenance of the TCA cycle and the mitochondrial electron transport in the conditions of an actively photosynthetic cell and decreased operation of the TCA cycle caused by the high level of ATP and reducing equivalents (Figure 7). They can serve as a basis for creating varieties/lines of crop plants with the increased expression of the genes encoding the enzymes of the GABA shunt—GDH, GAD, GTA, SSADH—which will be adapted to grow in regions with suboptimal or excessive sun irradiation. This will aim to expand the areas of arable land used in agriculture.

## 4. Materials and Methods

### 4.1. Object of Investigation

Leaves of two-week-old maize (*Zea mays* L., cv. Voronezhskaya-76 obtained from the Voronezh branch of the All-Russian Research Institute of Maize) seedlings were used in this study. Maize plants were germinated in water and grown hydroponically under 10 h daylight with the intensity of 90 µmol quanta m^−2^ s^−1^ in the climatic chamber “LabTech” (Namyangju, Republic of Korea) and ambient temperature of 25 °C.

### 4.2. Irradiation by Light of Different Wavelengths

Plants were placed in a chamber for 24 h (darkness option), after which they were irradiated with red (640–680 nm) and/or far-red (710–750 nm) light for 15 min, with the intensity of 4 µmol m^−2^ s^−1^, using, respectively, LEDs 640–680 nm (KIPD40M40-K-P6, Kaskad-Elektro, Moscow, Russia) and 710–750 nm (ZL127A-5-5, Kaskad-Elektro, Moscow, Russia). Samples for analysis were taken 3 h after irradiation. Additional incubation in the dark (3 h after irradiation) was necessary to trigger the mechanism of transduction of the light signal into the nucleus and obtain a response to this signal. To ensure that the change in enzyme activity was not related to the time of incubation in the dark, we used a dark control incubated in the dark (D) for 24 h. We also had an additional dark control of plants incubated in the dark for 27 h, which showed the same result as D, so we do not present it in the figures.

The exposure of plants to white light was carried out under normal conditions with a 12 h light incubation.

### 4.3. Treatment with EGTA and Ruthenium Red

To study the involvement of calcium in the regulation of GDH, two-week-old maize seedlings were incubated in darkness for 1 h with a 5 mM EGTA solution to bind Ca^2+^ ions or with ruthenium red (ammoniated ruthenium oxychloride, 25 µM) to block calcium channels or Ca-ATPases [35,76]. Following the treatment, the measurement of GDH activity in isolated mitochondria and the extraction of RNA for the study of the expression of GDH genes were performed.

### 4.4. Isolation and Assay of Glutamate Dehydrogenase

To determine GDH activity in the total cellular fraction, a sample of plant material was ground at +4 °C with the following extraction medium: 50 mM Tris-HCl (pH 8), 1 mM EDTA, 0.05% Triton X-100, 0.5% PVP-40. The homogenate was filtered, and the cell walls were precipitated at 5000× *g* for 3 min.

The mitochondrial fraction for the analysis of GDH activity in mitochondria was isolated by homogenizing the plant material in a porcelain mortar with the following isolation medium: 0.15 M Tris-HCl buffer (pH 7.4), 0.4 M sucrose, 2.5 mM EDTA, 1 mM KCl, 4 mM MgCl_2_, 0.05% Triton X-100 in a ratio of 1:10. The homogenate was filtered through four layers of cheesecloth and centrifuged for 3 min at 3000× *g* in an Eppendorf Centrifuge 5804 R (“Eppendorf”, Hamburg, Germany) to discard cell walls. The supernatant was centrifuged for 10 min at 18,000× *g*.

Cross-contamination of mitochondria by the cytosolic fraction was determined by analyzing the activity of alcohol dehydrogenase [77,78], while the degree of chloroplast contamination was assessed by analyzing the concentration of chlorophylls *a* and *b* [56,79]. The degree of cross-contamination of the cytoplasmic fraction did not exceed 4.5%, and chloroplast fraction—8%.

The isolated mitochondrial fraction was destroyed by osmotic shock in 0.15 M Tris-HCl buffer (pH 7.4). The degree of mitochondrial destruction was more than 90%, which was controlled by microscopy on an Olympus CX41RF (“Olympus”, Tokyo, Japan). The resulting mitochondrial fraction was used to determine GDH activity. All steps were carried out at a temperature of +4 °C.

Glutamate dehydrogenase (GDH; EC 1.4.1.3) activity in the amination reaction was determined spectrophotometrically at 340 nm in 0.1 M Tris-HCl buffer (pH 8.0) containing 13 mM 2-oxoglutarate, 0.25 mM NADH, 1 mM CaCl_2_, 50 mM (NH_4_)_2_SO_4_ [80]. The amount of enzyme that converts (aminates) 1 μmol 2-OG in 1 min at the optimal pH value was taken as a unit of GDH enzymatic activity.

### 4.5. Isolation and Assay of Glutamate Decarboxylase

Extraction of glutamate decarboxylase (GAD; EC 4.1.1.15) was performed by homogenizing plant material in 20 mM acetate buffer (pH 4.8) with the addition of 10 mM pyridoxal phosphate, followed by centrifugation at 12,000× *g* for 30 min. The supernatant was used to determine GAD activity spectrophotometrically by measuring the change in optical density of a solution containing 20 mM acetate buffer (pH 4.8), 70 μM bromocresol green, 10 mM pyridoxal-5-phosphate, 2 mM sodium glutamic acid at 620 nm for 3 min [81,82]. The amount of enzyme (GAD) that converts (decarboxylates) 1 μmol of glutamate in 1 min at the optimal pH value was taken as a unit of enzymatic activity.

### 4.6. Isolation and Assay of GABA Transaminase

Extraction of GABA transaminase (GTA; EC 2.6.1.19) was carried out by homogenizing leaves (1:10) in 50 mM Tris-HCl buffer (pH 8.5) containing 0.1 μM pyridoxal-5-phosphate, 0.05% Triton X100, and 20 μM β-mercaptoethanol, followed by centrifugation at 12,000× *g* for 30 min. GTA activity was determined in 50 mM Tris-HCl buffer (pH 8.5) containing 0.1 μM pyridoxal-5-phosphate, 5 mM 2-oxoglutarate, 200 μL of mitochondrial fraction, and 4 mM NAD^+^. The sample was incubated for 10 min at 25 °C, after which 16 mM GABA was added and the change in optical density was recorded at 340 nm [83]. The amount of enzyme that converts 1 μmol GABA in 1 min at 25 °C at the optimal pH value was taken as a unit of GTA enzymatic activity.

### 4.7. RNA Isolation and Reverse Transcription

To assess changes in the transcriptional activity of the *Gdh, Gad*, *Gta*, and *Ssadh* genes in maize leaves, we analyzed changes in the relative levels of their transcripts in real-time PCR using specific primers (Supplementary Table S1). The following genes were studied: the *Gdh1* (*LOC542220*) and *Gdh2* (*LOC100193614*) genes encoding the α and β subunits of GDH, the *Gad1* (LOC100381655) gene encoding GAD, the *Gta2* (*LOC103645944*) gene encoding GTA, and the *Ssadh1* (*LOC100280779*) and *Ssadh2* (*LOC100284047*) genes encoding SSADH. The efficiency of primers was estimated in the range of 0.2–0.8 μM by performing a series of dilutions followed by amplification by real-time PCR. The range of primer efficiency for the genes *Gdh1*, *Gdh2*, *Gad1*, *Gta2*, *Ssadh1*, and *Ssadh2* was 0.3–0.5 μM.

Total RNA was isolated by phenol/chloroform/isoamyl alcohol extraction using LiCl to remove DNA [84,85]. As a template for RT-PCR, we used cDNA obtained through a reverse transcription reaction with the MMLV-RT Kit (JSC Evrogen, Moscow, Russia) in accordance with the manufacturer’s protocol. The plant material (100 mg tissue per 1 mL medium) was ground in a porcelain mortar with an extraction medium containing 4 M guanidine thiocyanate, 30 mM sodium citrate, 30 mM β-mercaptoethanol, pH 7.0–7.5; then, 2 M sodium acetate (pH 4.0) was added (1/10 of the volume) and mixed, followed by adding 1 volume of phenol saturated with water. After vortexing, a mixture of chloroform and isoamyl alcohol (49:1) was added, mixed (20 s), and incubated on ice for 15–20 min. After centrifugation for 20 min at 10,000× *g* at 4 °C, the upper aqueous phase was transferred to a new Eppendorf tube and 1 mL of isopropanol was added, after which the mixture was incubated at −20 °C for 1 h. RNA was precipitated by centrifugation for 20 min at 10,000× *g* at 4 °C. The supernatant was removed, and the pellet was washed twice with 500 μL of 80% ethanol, dried, and dissolved in RNase-free water (100 μL).

### 4.8. Polymerase Chain Reaction

Real-time PCR was performed on a LightCycler96 device (Roche, Solna, Sweden) with the designed gene-specific primers (Supplementary Table S1), using SYBR Green I as an intercalating dye. Amplification was carried out according to the following parameters: preliminary denaturation—95 °C for 5 min, followed by 35 cycles, each containing the following steps: 95 °C—10 s, 57, 58, or 59 °C (see annealing temperature in the tables)—10 s, 72 °C—10 s. Finally, a 10 min final elongation was performed at 72 °C. Quantitative matrix control was carried out using gene-specific primers for housekeeping genes (*Ef-1α* elongation factor). Total RNA without the RT-PCR step was used as a negative control. Calculation of the relative levels of transcripts of the studied genes was performed using the 2^−∆∆Ct^ method [86].

### 4.9. Statistical Analysis

The number of plants within each group in each experiment was 6–14. The experiments were carried out in 3–4 biological replicates; analytical determinations for each sample were performed in triplicate. Statistical analysis of the obtained data was performed using the STATISTICA 12.0 program. Quantitative data were assessed for compliance with normal distribution using the Shapiro–Wilk test. The results in the graphs were expressed as the average ± standard error of the mean (SEM). Differences were analyzed for statistical significance using Student’s *t*-test with Bonferroni correction for multiple comparisons. Additionally, a one-way analysis of variance was used. The statistically significant differences (*p* ≤ 0.05) are discussed [87].

## 5. Conclusions

The results obtained in this study suggest that the irradiation of maize plants by light results in the suppression of GDH and activation of the enzymes participating in the GABA shunt. In most cases, this regulation occurs via the phytochrome mechanism, except for the second gene encoding SSADH. Phytochrome regulation, as shown for GDH, is mediated by concentration changes in Ca^2+^ ions. The induction of expression of the genes encoding the enzymes of the GABA shunt by light activates the bypass, leading to succinate production in the conditions when the metabolic flow through the 2-OGDH complex is suppressed. Thus, the GABA shunt is essential for the adaptation of plants to light conditions. Further studies of the mechanisms regulating the operation of enzymes involved in the GABA shunt will provide new insights into the epigenetic mechanisms of adaptation of plant cells to changes in the spectral composition of light.

## Data Availability

The datasets generated for this study are available upon request from the corresponding author.

## Supplementary Materials

The following supporting information can be downloaded at: https://www.mdpi.com/article/10.3390/ijms252312711/s1.

## Abbreviations

GABA	γ-aminobutyric acid
GAD	glutamate decarboxylase
GDH	glutamate dehydrogenase
GTA	GABA transaminase
2-OG	2-oxoglutarate
2-OGDH	2-oxoglutarate dehydrogenase
SSA	succinic semialdehyde
SSADH	succinic semialdehyde dehydrogenase
TCA cycle	tricarboxylic acid cycle
