# Supplementary material for: The Role of Glutamate Metabolism and the GABA Shunt in Bypassing the Tricarboxylic Acid Cycle in the Light
Source: Int J Mol Sci. 2024 Nov 26;25(23):12711. doi: 10.3390/ijms252312711 (PMC11641617; doi:10.3390/ijms252312711)
Supplement: Supplementary file 1 [file ijms-25-12711-s001.zip › ijms-3275115-supplementary.pdf]

Supplementary Figure and Table

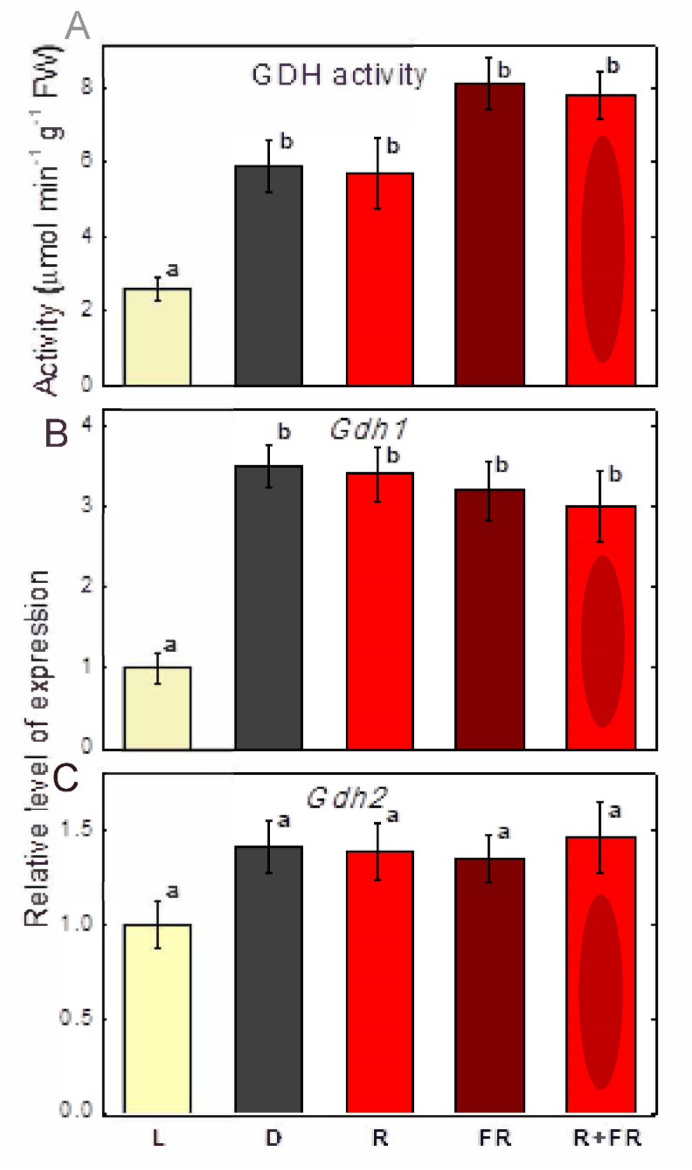

Supplementary Figure S1. Glutamate dehydrogenase (GDH) activity in maize leaf mitochondria and expression of its two genes *Gdh1* and *Gdh2* under different light conditions upon application of the calcium channels blocker ruthenium red to the seedlings. (**A**) – GDH activity in the mitochondrial fraction, (**B**) – Expression of the gene *Gdh1*, (**C**) – Expression of the gene *Gdh2*. L –light; D – dark; R – red light (660 nm); FR – far-red light (730 nm); R+FR – red light applied after far-red light. The results are presented as the average ± standard error of the mean (SEM). Differences were analyzed for statistical significance using Student's t-test with Bonferroni correction for multiple comparisons. The letters indicate statistically significant differences at $p < 0.05$ ($n = 5$).

Supplementary Table S1. Primers to the genes of glutamate dehydrogenase, glutamate decarboxylase, GABA transaminase, and succinic semialdehyde dehydrogenase

Gene	Primer	Oligonucleotide sequence	Annealing temperature, °C
Primers to the genes of glutamate dehydrogenase			
Gdh1	Forward	GCGGAGAACAAGGGGATCAA	58
	Reverse	ACAGGATCTCGTCTGCCTCT	
Gdh2	Forward	TGATCCAGAGGCAGACGAGA	58
	Reverse	GTAATGCGCGGTCAATGGTC	
Primers to the gene of glutamate decarboxylase			
Gad1	Forward	GGAGGCGTGGAAGAAGTTTG	59
	Reverse	CAACTCACTCGGTGCATAGC	
Primers to the gene of GABA transaminase			
Gta2	Forward	TCTACGGGGAAGCCCTCAAG	57
	Reverse	TTGAGCCACCATTTGCTTGG	
Primers to the genes of succinic semialdehyde dehydrogenase			
Ssadh1	Forward	CACAGCCTGGGGATGTCATT	58
	Reverse	GGTGCCACCTGCGTTTGTAT	
Ssadh2	Forward	GTGCTTAGAAGGGCGTGAGT	58
	Reverse	CAGGTTCGAAGGTGAAGCCA	
